# Developmental trajectories of anger and sadness dysregulation in childhood differentially predict later borderline symptoms

**DOI:** 10.1017/S0954579423000627

**Published:** 2023-06-21

**Authors:** Alecia C. Vogel, Ben Geselowitz, Rebecca Tillman, Deanna M. Barch, Joan L. Luby, Diana J. Whalen

**Affiliations:** 1 Department of Psychiatry, Washington University School of Medicine, St. Louis, MO, USA; 2 Department of Psychological and Brain Sciences, Washington University in St. Louis. St. Louis, MO, USA; 3 Department of Radiology, Washington University in St. Louis. St. Louis, MO, USA

**Keywords:** adolescence, borderline personality disorder, emotion awareness, emotion regulation, multilevel models

## Abstract

Difficulties with emotion regulation are integral to borderline personality disorder (BPD) and its hypothesized developmental pathway. Here, we prospectively assess trajectories of emotion processing across childhood, how BPD symptoms impact these trajectories, and whether developmental changes are transdiagnostic or specific to BPD, as major depressive (MDD) and conduct disorders (CD) are also characterized by emotion regulation difficulties. This study included 187 children enriched for those with early symptoms of depression and disruptive behaviors from a longitudinal study. We created multilevel models of multiple components of emotional processing from mean ages 9.05 to 18.55 years, and assessed the effect of late adolescent BPD, MDD, and CD symptoms on these trajectories. Linear trajectories of coping with sadness and anger, and quadratic trajectories of dysregulated expressions of sadness and anger were transdiagnostic, but also exhibited independent relationships with BPD symptoms. Only inhibition of sadness was related to BPD symptoms. The quadratic trajectories of poor emotional awareness and emotional reluctance were also independently related to BPD. Findings support examining separable components of emotion processing across development as potential precursors to BPD, underscoring the importance of understanding these trajectories as not only a marker of potential risk but also potential targets for prevention and intervention.

## Introduction

Borderline personality disorder (BPD) is a relatively common and highly impairing diagnosis characterized by affective lability and impulsivity, as well as unstable interpersonal relationships and self-image (American Psychiatric Association, 2013). BPD affects 1–2% of the general population (Torgersen et al., 2001), but prevalence rises to 20% in patients receiving outpatient psychiatric care (Korzekwa et al., 2008) and to 50% in patients receiving inpatient care (Grilo et al., 1998). BPD is associated with severe functional impairment across several domains, including a rate of suicide almost 50 times higher than the general population (Pompili et al., 2005), educational and occupational morbidity, and extensive health services utilization (van Asselt et al., 2007). Yet, research on developmental precursors leading to BPD lags far behind other disorders such as major depressive disorder (MDD) and externalizing pathology. Additional work is greatly needed that addresses both shared and unique developmental precursors to BPD that help to differentiate developmental trajectories of BPD from other common and co-occurring disorders (Stepp, Lazarus, et al., 2016).

Historically, BPD has been viewed as a disorder of adulthood. However, recent work challenges this assumption and recognizes the developmental nature of BPD and associated precursors beginning during childhood. BPD is theorized to develop from interactions between biological vulnerabilities and environmental risk factors (see reviews in Beauchaine et al., 2009 and Crowell et al., 2009). Similar to other diathesis–stress models of psychiatric illness, developmental theories of BPD (Crowell et al., 2009) based in the biosocial model (Linehan, 1993) argue that biological vulnerabilities, such as impulsivity, interact with environmental effects, such as coercive and invalidating environments, to produce increasingly dysregulated emotional responses which interact with interpersonal and identity processes to culminate in BPD psychopathology. An increasing number of studies have supported this model by assessing developmental precursors leading to BPD symptoms and diagnostic onset (for a review see Stepp, Lazarus, et al., 2016). For instance, there are numerous studies demonstrating associations between impulsivity (Fischer et al., 2002; Fossati et al., 2015; Miller et al., 2008; O’Grady & Hinshaw, 2023; Stepp et al., 2012), emotional sensitivity and reactivity (Stepp, Scott, et al., 2016), and later development of BPD. Additional lines of work have found that developmental stressors, including invalidating caregiving and hostility (Musser et al., 2018), disorganized attachment (Mosquera et al., 2014), childhood trauma (Bornovalova et al., 2013), and peer victimization (Winsper et al., 2017) also correspond to later BPD symptoms and diagnosis. Despite this burgeoning empirical literature, reviews and meta-analyses repeatedly indicate that these developmental precursors are largely nonspecific (Bozzatello et al., 2020; Gunderson et al., 2018; Hutsebaut & Aleva, 2021; Stepp, Lazarus, et al., 2016; Stepp & Lazarus, 2017; Winsper et al., 2017).

Thus, in the current paper, we focus on multifinality by examining shared and unique aspects of trajectories of emotion dysregulation as precursors of BPD, depression, and/or externalizing pathology (specifically conduct disorder). Emotion dysregulation has been extensively phenotyped across psychiatric presentations (Beauchaine et al., 2009; Beauchaine, 2015; Beauchaine & Zisner, 2017; Chapman, 2019; Crowell & Kaufman, 2016; Gross & Jazaieri, 2014; Vlisides-Henry et al., 2021). However, emotion dysregulation is theorized to be a core and central contributor to the development and maintenance of BPD (Chapman, 2019; Crowell et al., 2009; Putnam & Silk, 2005). While disturbances in self and social processing are also critical to BPD development, emotional dysregulation with intense expressions of distress is the most widely studied feature (for review see Chapman, 2019). In fact, emotion dysregulation is a salient risk factor for nonsuicidal self-harm and suicidality, some of the most consequential and damaging complications of BPD (Anestis et al., 2011; Beauchaine et al., 2019; Rajappa et al., 2012; Wolff et al., 2019). In adults, research and clinical observations support a relationship between problems with general difficulties in emotion regulation and BPD (Kuo & Linehan, 2009), as well as specific relationships between BPD and poor distress tolerance (Gratz et al., 2006), higher emotional avoidance (Beblo et al., 2013), as well as lower emotional awareness (Leible & Snell, 2004).

In the biosocial model of BPD development, impulsivity and emotional lability interact with environmental factors, leading to increasingly dysregulated emotional expressions. In keeping with this model, difficulties in emotion regulation in young adults partially mediate the relationship between affective intensity and borderline symptoms (Salsman & Linehan, 2012). Difficulties with emotion regulation also partially mediate the relationship between childhood trauma and BPD symptoms (Kuo et al., 2015; Peng et al., 2021). More specifically, the relationship between retrospective assessments of negative familial emotional interactions or parental coercion and BPD symptoms was mediated by emotion regulation (Hope & Chapman, 2019; Kaufman et al., 2017). When coercive and invalidating parenting is directly assessed via observation, maternal invalidation increased children’s emotional expressions of anger in those with BPD traits (Crowell et al., 2013). Subsequent work demonstrated this is a transactional process in dyads with self-injuring youth, where their dysregulation increased maternal hostility which then increased adolescent anger (Crowell et al., 2017).

However, despite their utility, these studies have either relied on retrospective reports of environmental effects or the analysis of cross-sectional assessments in environmental interactions, emotion dysregulation, and BPD symptoms. In a prospective, longitudinal study of emotion processing in children with BPD symptoms, the baseline severity of BPD symptoms predicted emotion dysregulation, and the severity and trajectory of emotion dysregulation then predicted subsequent BPD symptoms; the trajectory of emotion dysregulation mediated the relationship between baseline and BPD symptoms at later follow-up (Stepp, Scott, et al., 2014). In work by Carlson et al., not only did early childhood activity level and caregiver factors relate to BPD symptoms in adulthood, but middle childhood behavioral and emotion instability, indicators of emotion dysregulation, did as well (Carlson et al., 2009). Such work emphasizes the longitudinal nature of these relationships and the importance of studying the developmental unfolding of emotion dysregulation prospectively and before disorder onset.

Originating in childhood, BPD can be conceptualized via multifinality: early, nonspecific risk factors such as impulsivity and emotional lability interact with environmental factors to influence the trajectory of emotion regulation/dysregulation across development. Thus, the overall trajectory of emotion regulation (and dysregulation) plays a key role in the development and persistence of BPD symptomatology. However, current data is largely limited to assessing these relationships cross-sectionally or using retrospective assessments. The entirety of childhood, and particularly the early developmental period, is rarely considered and/or measured. For instance, our own prior work shows BPD precursors can be identified as early as preschool (Geselowitz et al., 2020), and prior work by Carlson et al., has found relationships between both child and family factors in infancy and childhood with adult BPD symptoms (Carlson et al., 2009). In our prior work, we demonstrated unique relationships between preschool internalizing and externalizing symptoms, low levels of observed maternal support, preschool adverse childhood experiences, and adolescent BPD symptoms (Geselowitz et al., 2020). Thus, the trajectory of risk for BPD should be assessed across childhood, even from the youngest ages. In the current paper, using our 17-year longitudinal study, we assess how the trajectory of emotion regulation from childhood through adolescence impacts the onset of adolescent BPD symptoms.

Moreover, prior studies have largely considered emotion regulation or dysregulation as a single entity, grouping negative emotions such as a sadness and anger, as well as adaptive and potentially maladaptive regulation strategies such as inhibition, and maladaptive expressions of emotion. Descriptions of an invalidating environment (Crowell et al., 2009, 2013; Kuo & Linehan, 2009; Musser et al., 2018) specify an environment in which the caregiver eschews or rejects the child’s emotional reactions and intermittently reinforces extreme expressions of negative affect. We hypothesize that children experiencing such an environment may be more likely to both generally inhibit their emotions or have expressional reluctance, as well as have increased dysregulated expressions of emotion. However, we are unaware of other studies assessing these components separately, particularly during childhood before disorder onset. Additionally, grouping emotions can be useful, as there is often a moderately strong relationship between dysregulated expressions of various emotions (Suveg et al., 2009). However, children (Waters & Thompson, 2014) and adolescents (Zimmermann & Iwanski, 2014) report using different emotion regulation skills for sadness and anger. Further, the diagnostic criteria for BPD specifically references “inappropriate, intense anger or difficulty controlling anger” (American Psychiatric Association, 2013). Yet, as anger has not been assessed separately from other negative emotions such as sadness, it is unclear if expressions and/or inhibition of anger across development are more closely related to BPD, relative to sadness.

Given the importance of emotion dysregulation in BPD, along with the expected changes in emotion regulation occurring during typical development, studying longitudinal trajectories of emotion regulation can inform our understanding of how BPD develops and perhaps, how this may differentiate the development of BPD from other related disorders such as depression and externalizing pathology. Indeed, depression often precedes the development of BPD (Bohus et al., 2021; Stepp, Scott, et al., 2016; Stepp & Lazarus, 2017). Developmental work has also illuminated risk trajectories for BPD emerging from childhood externalizing pathology (Crowell et al., 2009; Hallquist et al., 2015; Lyons-Ruth & Brumariu, 2021; Stepp et al., 2012; Stepp, Whalen, et al., 2014; Underwood et al., 2011; Wright et al., 2016). However, there is little empirical data that has examined developmental trajectories of emotion dysregulation leading to all three outcomes (although see Beeney et al., 2021 for an excellent example), and it is unclear what aspects of emotion regulation and dysregulation trajectories are unique to BPD versus depression and externalizing pathology, a crucial gap given the unique intervention strategies and prognoses for these disparate outcomes.

Understanding the developmental trajectory of various components of emotion processing may improve our ability to change those trajectories. While there are effective psychotherapeutic interventions for BPD (Linehan et al., 2006), including in adolescents (McCauley et al., 2018), these remain relatively time intensive with limited availability and high cost. Understanding whether there are particular components of dysregulation that are most influential in the development of BPD may help specify treatment targets prior to disorder onset and ensuing impairment. Additionally, interventions are often not initiated until long after the onset of impairing symptoms, given that the average age of first treatment for BPD is 18 (Zanarini et al., 2001), while the onset of self-injury begins prior to age 18 in as many as two thirds of BPD patients (Zanarini et al., 2006). For example, Crowell and colleagues (2013) found that adolescents (ages 13–17 years) with self-harm scored higher on measures of BPD pathology. The trajectory of BPD symptoms in adolescence also predicts the development of social skills, sexual activity, and self-perception (Wright et al., 2016). Thus, continuing to improve our ability to identify risk trajectories for BPD earlier in childhood may help direct treatment to the most at-risk youth.

Here, we assess the longitudinal trajectories of emotional processing (regulation and dysregulation) from middle childhood through adolescence in a group of children with early-onset psychopathology, who have exhibited elevated rates of BPD symptoms in late adolescence (Boone et al., 2022; Geselowitz et al., 2020). Not only were these emotional processing measures prospectively gathered, but they include separable scales for emotional coping, dysregulation, and inhibition assessed individually for sadness and anger, along with additional scales measuring emotional reluctance and awareness. This allows us to test: (1) whether the developmental trajectories of emotional processing generally impact BPD development; and (2) whether any relationship is specific to emotional dysregulation, inhibition and reluctance, and more specific to anger, as hypothesized. Additionally, as these children were also at increased risk for depression, we were able to assess if there is a specific relationship between developmental trajectories and BPD versus depression and externalizing pathology.

## Methods

### Participants

**Figure 1.**
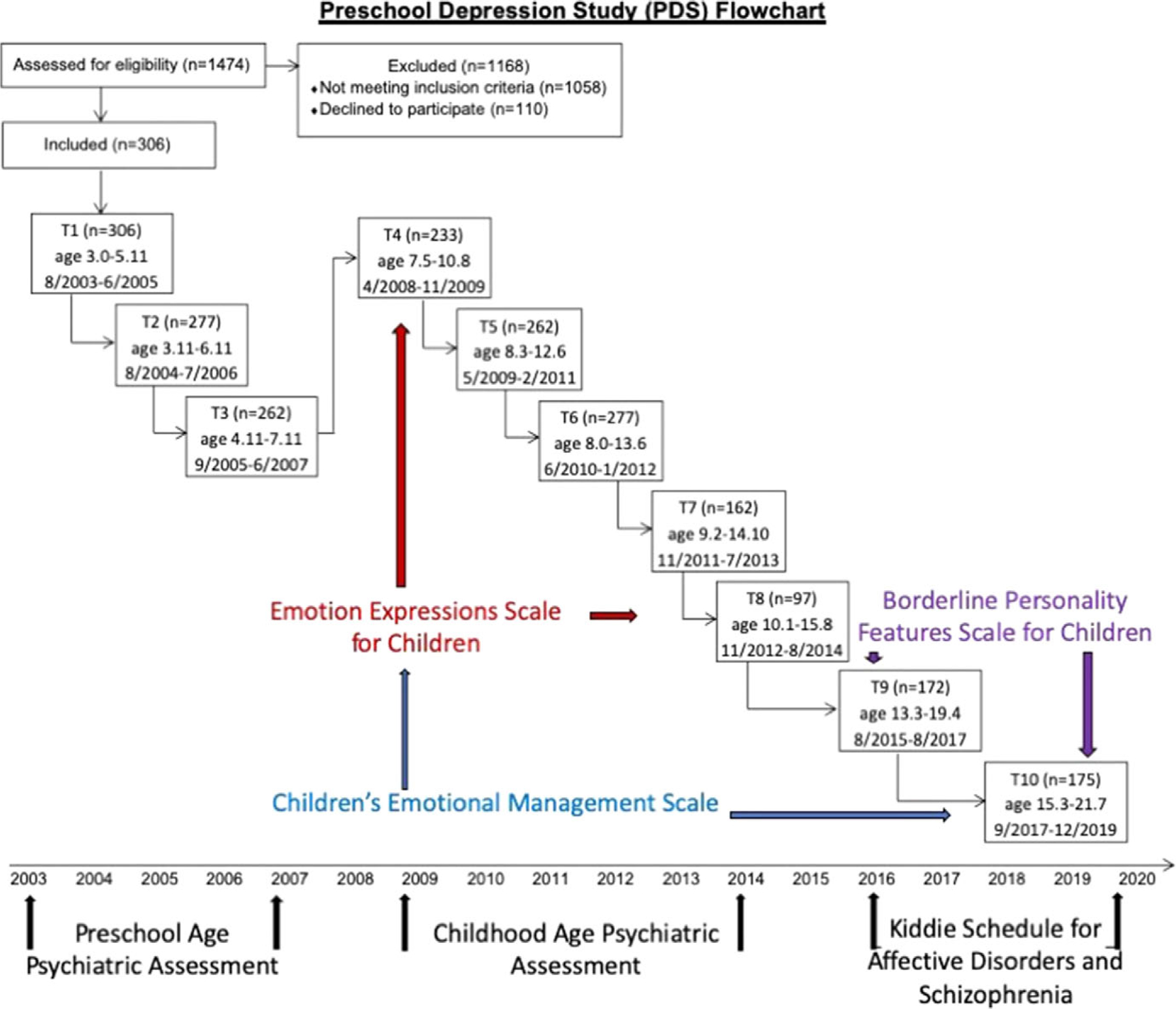
Preschool depression study flowchart.

Participants were enrolled in the Preschool Depression Study (PDS), a prospective, longitudinal investigation of preschoolers and their families conducted at the Washington University School of Medicine Early Emotional Development Program that has been extensively described elsewhere (Luby et al., 2014). Initially, 306 3–6-year-old children and their caregivers were recruited from primary care clinics and day care centers in the St Louis Metropolitan area oversampling for depression using the Preschool Feelings Checklist (Luby et al., 2004), a validated measure assessing depressive symptoms in the preschool age. Children with symptoms of disruptive behaviors and typically developing children were recruited as comparison groups. All were invited to participate in up to an additional five assessments including clinical interviews, observational assessments, and behavioral questionnaires. A subset of these children were invited for five neuroimaging scans and additional behavioral assessments at those times. In total, assessments spanned 17 years, and a study summary is depicted in Figure 1. All components of this study were done in accordance with review and approval from the Washington University School of Medicine Institutional Review Board (IRB# 201502094). Participants and caregivers were compensated for their time. At each wave, parents provided informed consent; children provided oral consent for the first eight assessments, written assent at the final two assessments (when participants were ages 13.3–21.1 years) if they were not yet 18 years, and informed consent when reaching age 18 years.

The present study includes 187 participants who completed at least one middle childhood/adolescent timepoint (assessments 4–10, median age 9.0–18.8 years), which allowed for the calculation of emotion regulation trajectories, and who also completed the Borderline Personality Features Scale for Children (BPFS-C, Crick et al., 2005) during assessment 9 or 10 (median age 16.3 and 18.8). Demographic information can be found in Table 1. There were no significant differences in demographics or outcome variables between those children included versus excluded from the analyses (Supplemental Table S1).

### Measures

#### Borderline personality symptoms

BPD symptoms were measured by the Borderline Personality Features Scale for Children (BPFS-C, Crick et al., 2005) at assessments 9–10 (median age 16.3–18.8). The BPFS-C is a self-report measure with established construct validity (Crick et al., 2005) and high criterion validity (Chang et al., 2011). Scores range from 24 to 120, with higher scores indicating more BPD symptoms; a score greater than 65 is considered the clinical cutoff for a presumptive BPD diagnosis (Chang et al., 2011). Scores on the BPFS-C converge with interview-based measures of BPD during adolescence (Chang et al., 2011). For participants who had measures at both timepoints, a mean score was calculated.

### Emotion processing scales

Emotion regulation was measured by the Child Emotional Management Scales-Anger and -Sadness (CEMS-A and CEMS-S, (Zeman et al., 2001, 2002), collected at assessments 4–10 (median age 9.0–18.8). The CEMS-A and CEMS-S are widely used, validated, and reliable self- and parent-report measures of children’s emotion regulation strategies in response to anger and sadness. Here, we utilize the self-report measures. The CEMS-A and CEMS-S have been shown to be related to other measures of emotion regulation (Zeman et al., 2001) and symptoms of psychopathology (Zeman et al., 2002). Recent work confirms the original three-factor structure (coping, dysregulation, and inhibition) in a diverse, psychiatric sample (Ogbaselase et al., 2022). The dysregulation and inhibition subscales, which assess use of maladaptive coping skills, as well as the coping subscale, which assesses use of adaptive coping skills, were all considered separately.

The Emotion Expression Scale for Children (EESC, Penza-Clyve & Zeman, 2002), a self-report questionnaire with high internal consistency and moderate test–retest reliability that examines lack of emotion awareness and lack of motivation to express negative emotion (Penza-Clyve & Zeman, 2002), was administered at assessments 4–8 (median age 9.0–13.4). Here, we included both the poor awareness and expressive reluctance subscales. There are moderate and significant correlations between many of the baseline emotion processing assessments (Table 2), however the strongest correlation was 0.672 between the two EESC subscales, supporting our decision to analyze the effect of each emotion processing scale independently.

### Psychiatric symptoms

The core symptoms of MDD and diagnosis were measured via the Preschool Age Psychiatric Assessment (PAPA, Egger & Angold, 2004) from baseline to assessment 3, the Childhood and Adolescent Psychiatric Assessment (CAPA, Angold & Costello, 2000) at assessments 4–8, and the Kiddie Schedule for Affective Diagnosis and Schizophrenia (KSADS, Kaufman et al., 2016) at assessments 9–10. Conduct disorder (CD) symptoms and diagnosis at assessments 9–10 was measured via KSADS (Kaufman et al., 2016).

### Adverse childhood experiences

The Z-transformed Adverse Childhood Experiences Score (ACES-Z) was based on items identified by Felitti et al. (1998), summing items assessing parent-reported poverty, parent-reported parental suicide attempts, substance abuse, and psychopathology from the Family Interview for Genetic Studies (FIGS), and parent- or child-reported traumatic events from the PAPA (assessments 1–3), CAPA (assessments 4–8), and Life Events Checklist (assessments 9–10). This total was converted to a Z-score at each wave and averaged across waves during the preschool period (age 3.0–5.11) and overall (assessments 1–10), as detailed elsewhere (Barch et al., 2018).

### Questionnaire completion and informants

Table 3 provides details about the number of participants completing each of the measures and participant age by wave. For all caregiver-reported measures (e.g., the psychiatric interviews and FIGS), we obtained information from the family’s identified caregiver. In 94% of cases, the primary caregiver identified was the child participant’s mother.

### Analyses

Multilevel models (MLM’s) were used to investigate the association of BPFS-C scores (as an independent variable) with each of the coping, dysregulated, and inhibited expression subscales of the CEMS-A and -S, as well as the poor awareness and expressive reluctance subscales of the EESC (in individual models as the dependent variable). Each model included the effect of time as measured by years after the first study wave at which CEMS or EESC was administered, the participant’s age at that first wave, ACES-Z as assessed as above, sex as reported by parent at baseline, family history of affective diagnosis, and BPFS-C score. To assess whether linear or nonlinear models provided the best fit for the data, models were run with time, time-squared, and time-cubed effects. For those models in which there were significant quadratic and/or cubic effects, likelihood ratio tests were conducted to determine which of the nested models provided the best fit, and that model was chosen. In such models, the independent variables, including BPFS-C, could influence not only the overall level of emotion processing (i.e., have the same trajectory shape and slope, just moved up or down from the average), but may also alter the trajectory of emotion processing (i.e., have a steeper slope in emotion processing development at higher rates of BPD symptoms). To assess whether such interactive effects were present and BPD symptoms explained variance in the trajectories of emotion processing measures over time, we included a BPFS-C × time interaction, which was retained in the final model whenever significant. Given that we defined 8 different MLMs, results were corrected for multiple comparisons using the false discovery rate (FDR, Benjamini & Hochberg, 1995). Specifically, all models were run including the BPFS-C × time interaction, and the interaction *p*-values were FDR corrected. Then, the interaction was removed from all models, and the main effect of BPFS-C was FDR corrected.

When individual items on the CEMS or EESC measures were missing, subscales were calculated by replacing the missing value with the mean value for that item among age- and gender-matched subjects. Therefore, there were no subjects with missing CEMS or EESC subscale scores at waves these measures were completed. Only participants who completed the BPFS-C at assessment 9 and/or 10 and had nonmissing data for the other variables included in the models were included in the analyses. MLM’s allow for missing assessment waves but require full data at each completed wave, so only waves at which the CEMS or EESC were completed contributed to the model, and participants with at least 1 wave of data were included.

Similar MLM’s substituting categorical BPD (defined as BPFS-C > 65 at assessment 9 or 10) for continuous BPFS-C score, were performed, also controlling for sex, ACES-Z, and lifetime MDD diagnosis. These models were also corrected for multiple comparisons (Benjamini & Hochberg, 1995) and are reported in the supplement.

Last, we assessed the specificity of these developmental trajectories of emotion regulation to the development of BPD symptoms relative to symptoms of depression and CD, as justified in the introduction. Similar MLM analyses assessing the relationships between emotion regulation measures and symptoms of MDD and CD were performed. Results were corrected for multiple comparisons using FDR (Benjamini & Hochberg, 1995).

All analyses were conducted using SAS v9.4.

## Results

Models assessing the developmental trajectories of coping with negative emotions, dysregulated expressions of negative emotions, and inhibition of negative emotions including anger and sadness and their relationship to BPD are shown in Table 4. We detail each of the findings below. To quantify the multifinal effects of these development trajectories of emotion processing, we assessed their relationships to MDD and CD as well (Table 5), with specific relationships discussed below.

### Emotion coping

As expected, there is an increase in self-ratings of both anger and sadness coping over time, which are both best described by linear models, and there was also a significant effect of age at study entry (Estimate = 0.19, SE = 0.07, *p* = 0.0063) and female sex (Estimate = −0.55, SE = 0.16, *p* = 0.0008) on sadness coping trajectory.

**Figure 2.**
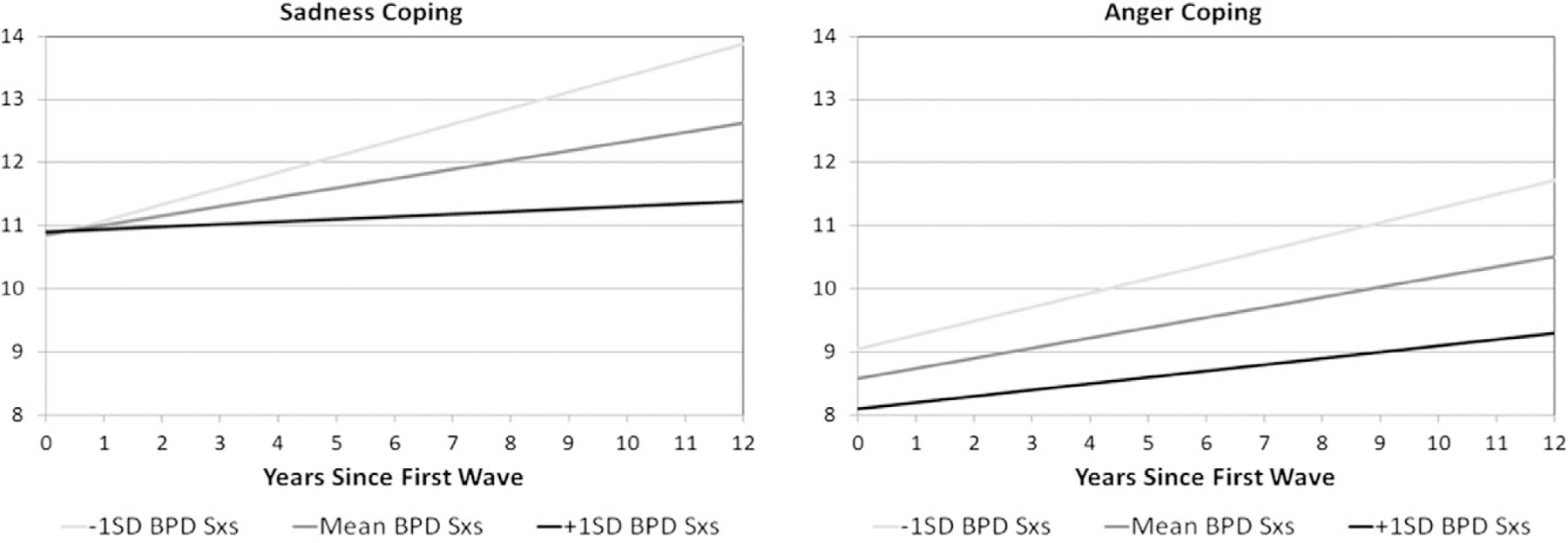
Estimated trajectories of emotion coping over time. *Note*. BPD Sxs = borderline personality disorder symptoms; Trajectories were assessed via CEMS-sadness and - anger self-report scales. Trajectories are plotted for the mean BPFS-C score as well as those 1 SD above the mean and 1 SD below the mean, shaded by intensity of BPFS-C symptoms.

Notably, there is an interaction between BPD symptoms and time explaining a small but significant proportion of the variance in the trajectory of both anger (Estimate = −0.005, SE = 0.001, FDR *p* = 0.0036) and sadness (Estimate = −0.008, SE = 0.001, FDR *p* < 0.0001) coping scores, indicating different changes in trajectories of coping over time at different levels of BPD symptoms. Graphs of estimated trajectories demonstrate that those with more BPD symptoms have a slower increase in coping over time than those with fewer BPD symptoms (Figure 2). In addition to differences in trajectories of anger coping over time by number of BPD symptoms, there is also a significant difference in anger coping scores at the first wave (Estimate = −0.038, SE = 0.010, *p* = 0.0001), indicating decreased anger coping in those with more symptoms of BPD.

When late adolescent MDD and CD symptoms were substituted for BPD symptoms in separate models, these also had significant effects (Table 5). There was a significant effect of the interaction between MDD symptoms and time on coping with sadness (Estimate = −0.037, SE = 0.009, FDR *p* < 0.0001) and a marginally significant effect with anger (Estimate = −0.021, SE = 0.009, FDR *p* = 0.0523). However, when MDD and BPD symptoms were both included in the same model with covariates, BPD symptoms were independently associated with coping symptoms while MDD symptoms were not (*Sadness coping-* BPD × time: Estimate = −0.008, SE = 0.001, *p* < 0.0001, MDD: Estimate = −0.002, SE = 0.046, *p* = 0.9590; *Anger coping*- BPD × time: Estimate = −0.005, SE = 0.001, *p* = 0.0007, MDD: Estimate = 0.009, SE = 0.049, *p* = 0.8500). The interaction between CD symptoms and time also had a significant effect on both sadness (Estimate = −0.079, SE = 0.025, FDR *p* = 0.0144) and anger (Estimate = −0.071, SE = 0.024, FDR *p* = 0.0148) coping. However, when CD and BPD symptoms were both included in the same model with covariates, BPD symptoms were independently associated with coping symptoms while CD symptoms were not (Sadness coping- BPD × time: Estimate = −0.008, SE = 0.001, *p* < 0.0001, CD: Estimate = −0.030, SE = 0.116, *p* = 0.7995; Anger coping- BPD × time: Estimate = −0.005, SE = 0.001, *p* = 0.0008, CD: Estimate = −0.234, SE = 0.123, *p* = 0.0583).

### Emotion dysregulation

Sadness and anger dysregulation demonstrate a more complicated developmental profile. Quadratic models provided the best fit for sadness and anger dysregulation ratings across time. There was a general decrease in ratings of sadness and anger dysregulation in the first several assessments (after the initial baseline assessment) followed by a subsequent increase at the later assessments.

**Figure 3.**
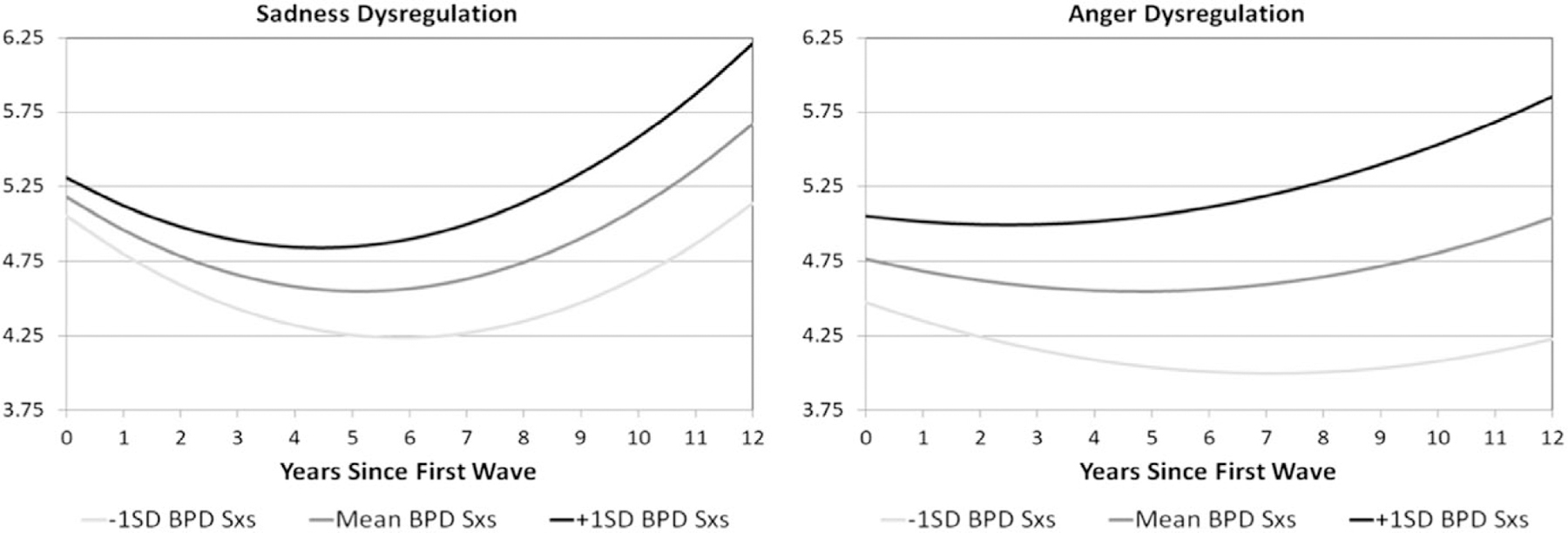
Estimated trajectories of emotion dysregulation over time. *Note*. BPD Sxs = borderline personality disorder symptoms; Dysregulation was assessed via the self -eport CEMS-sadness and -anger and dysregulation scales. Trajectories are plotted for the mean BPFS-C score as well as those 1 SD above the mean and 1 SD below the mean, shaded by intensity of BPFS-C symptoms.

Similar to the coping trajectories, there is an interaction between BPD symptoms and time explaining a small but significant proportion of the variance in the trajectory of both sadness (Estimate = 0.003, SE = 0.001, FDR *p* = 0.0234) and anger (Estimate = 0.003, SE = 0.001, FDR *p* = 0.0040) dysregulation scores, indicating different changes in trajectories of dysregulation over time at different levels of BPD symptoms. Graphs of estimated trajectories demonstrate that those with more BPD symptoms have less decrease in dysregulation immediately following the first wave with a steeper increase over the later waves relative to those with fewer BPD symptoms (Figure 3). In addition to differences in trajectories of anger dysregulation over time by number of BPD symptoms, there is also a significant difference in anger dysregulation scores at the first wave (Estimate = 0.024, SE = 0.007, *p* = 0.0015), indicating increased anger dysregulation in those with more symptoms of BPD.

When late adolescent MDD and CD symptoms were substituted for BPD symptoms in separate models, there was a significant effect of MDD symptoms only on anger dysregulation (Estimate = 0.118, SE = 0.038, FDR *p* = 0.0096) (Table 5). There was no effect of MDD symptoms or the MDD × time interaction on sadness dysregulation. Similar to the coping models, when MDD and BPD symptoms were both included in the same model with covariates, BPD symptoms were independently associated with anger dysregulation while MDD symptoms were not (BPD × time: Estimate = 0.003, SD = 0.001, *p* = 0.0012, MDD: Estimate = 0.033, SE = 0.038, *p* = 0.3830). Similar to the MDD models, the interaction between CD symptoms and time was significantly associated with anger dysregulation (Estimate = 0.046, SE = 0.018, FDR *p* = 0.0317), but there was no significant effect of CD symptoms or the CD × time interaction on sadness dysregulation. When CD and BPD symptoms were both included in the same model, BPD symptoms were independently associated with anger dysregulation while CD symptoms were not (BPD × time: 0.003, SE = 0.001, *p* = 0.0012, CD: Estimate = 0.050, SE = 0.097, *p* = 0.6028).

### Emotion inhibition

**Figure 4.**
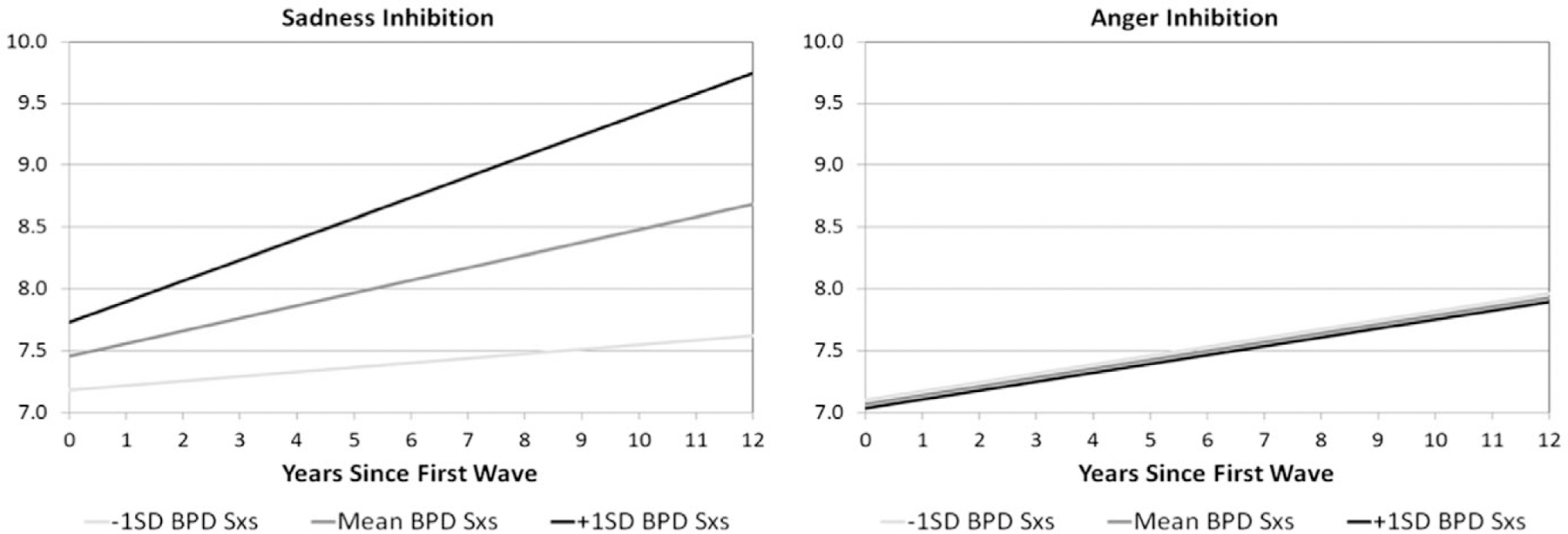
Estimated trajectories of emotion inhibition over time. *Note*. BPD Sxs = borderline personality disorder symptoms; Emotion inhibition was assessed via the self-report CEMS-sadness and -anger scales. Trajectories are plotted for the mean BPFS-C score as well as those 1 SD above the mean and 1 SD below the mean, shaded by intensity of BPFS-C symptoms.

Unlike the dysregulation and coping scales, inhibition of anger and sadness differed in their relationship to BPD symptoms. Both anger and sadness increased over time and were best described by linear models. Interestingly, there was no relationship between BPD symptoms and the trajectory of anger inhibition, so the interaction was removed from the final model. However, the interaction of BPD symptoms and time accounted for a significant amount of variance in sadness inhibition (Estimate = 0.005, SE = 0.002, FDR *p* = 0.0048), with graphs of estimated trajectories demonstrating those with more BPD symptoms having sharper increases in sadness inhibition over time relative to those with fewer BPD symptoms (Figure 4). Additionally, BPD symptoms also account for variance in sadness inhibition at the first wave (Estimate = 0.022, SE = 0.009, *p* = 0.0168), indicating higher initial rates of sadness inhibition in those with increased BPD symptoms. As with other components of expressions of sadness, female sex also partially accounted for variance in the trajectory (Estimate = −0.539, SE = 0.168, *p* = 0.0016).

When late adolescent MDD and CD symptoms were substituted for BPD symptoms in the model, there was a significant effect of MDD symptoms × time only on sadness inhibition (Estimate = 0.030, SE = 0.010, FDR *p* = 0.0124) (Table 5). CD symptoms had no significant effect on sadness inhibition. As with BPD symptoms, neither MDD nor CD symptoms had an effect on anger inhibition. Similar to the above, when MDD and BPD symptoms were both included in the same model with covariates, BPD symptoms were independently associated with sadness inhibition while MDD symptoms were not (BPD × time: Estimate = 0.005, SE = 0.002, *p* = 0.0028, MDD: Estimate = 0.063, SE = 0.048, *p* = 0.1886).

### Emotional expression

**Figure 5.**
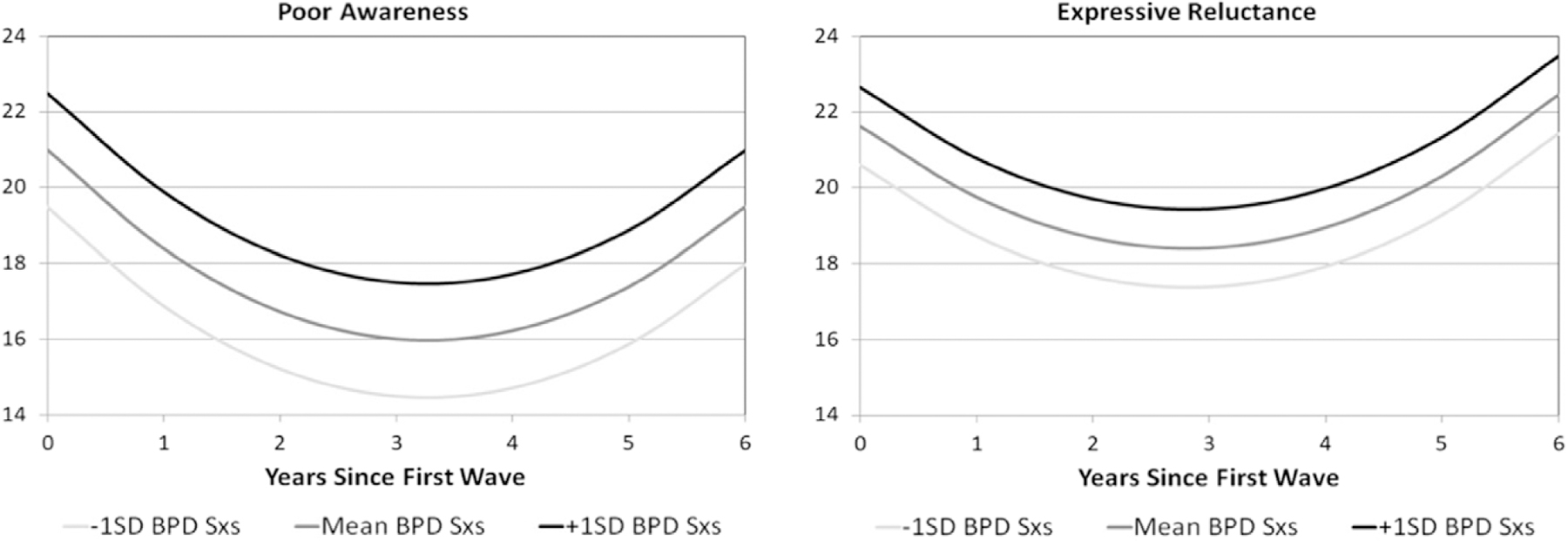
Estimated trajectories of poor emotional awareness and expressive reluctance over time. *Note*. BPD Sxs = borderline personality disorder symptoms; Emotional awareness was assessed via the poor awareness subscale of the EESC and expressive reluctance via the EESC subscale of the same name. Trajectories are plotted for the mean BPFS-C score as well as those 1 SD above the mean and 1 SD below the mean, shaded by intensity of BPFS-C symptoms.

Quadratic models provided the best fit for both poor emotional awareness and expressive reluctance. There was a decrease in both scores during the first few assessments (after the baseline assessment) that increased again during later study assessments. BPD symptoms again explain small but significant proportions of the variance in both trajectories (poor awareness: Estimate = 0.121, SE = 0.029, FDR *p* < 0.0001; expressive reluctance: Estimate = 0.087, SE = 0.025, FDR *p* = 0.0006). Graphs of estimated trajectories demonstrate those with more BPD symptoms had overall higher “poor awareness” scores and overall higher expressive reluctance than those with fewer BPD symptoms (Figure 5). There was an additional effect of age at first assessment on the trajectory of poor emotional awareness scores (Estimate = −0.78, SE = 0.30, *p* = 0.0113).

When late adolescent MDD and CD symptoms were substituted for BPD symptoms in separate models, there was a similar effect of MDD symptoms × time on both poor awareness (Estimate = 0.528, SE = 0.180, *p* = 0.0101) and a main effect of MDD symptoms on expressive reluctance (Estimate = 0.346, SE = 0.153, *p* = 0.0390) (Table 5). CD symptoms had no significant effect on either. Again, when MDD and BPD symptoms were both included in the same model with covariates, BPD symptoms were independently associated with poor awareness (BPD: Estimate = 0.101, SE = 0.032, *p* = 0.0018, MDD: Estimate = 0.300, SE = 0.194, *p* = 0.1235) and expressive reluctance (BPD: Estimate = 0.076, SE = 0.027, *p* = 0.0062, MDD: Estimate = 0.168, SE = 0.166, *p* = 0.3144), while MDD symptoms were not.

## Discussion

In this group of participants enriched for early childhood psychopathology, we find developmental changes in various facets of negative emotional expression across childhood, which relate to BPD symptoms. However, there are notable differences between the developmental trajectories of coping with negative emotions, the dysregulated expressions of negative emotions, and the inhibition of negative emotions. Moreover, there are interesting differences between the developmental trajectories of anger and sadness in their relationship to BPD (Table 4).

The developmental trajectory of emotion dysregulation is a key aspect of the theoretical pathway to BPD in biosocial models, and prior literature demonstrates that dysregulated expressions of emotions are a key mediator between early markers of risk, such as impulsivity and childhood abuse, and BPD symptoms. Here, we use longitudinal modeling to demonstrate that the trajectories of specific components of emotion regulation from childhood to adolescence are associated with adolescent BPD symptoms. Specifically, we show that increased BPD symptoms correspond with a slower improvement in coping with sadness and anger, greater dysregulated expressions of sadness and anger, a faster increase of inhibition of sadness, and greater emotional reluctance and poor awareness. Interestingly, we found no relationship between the developmental trajectory of anger inhibition and BPD symptoms. More gradual developmental trajectories of coping with negative emotions appears to be transdiagnostic, as these showed similar relationships to late adolescent MDD and CD symptoms. In contrast, there was a relatively specific relationship between the trajectory of sadness dysregulation and BPD symptoms relative to MDD or CD symptoms. We found similar associations between late adolescent BPD and MDD symptoms with anger dysregulation, sadness inhibition, poor emotional awareness and expressive reluctance, however the variance explained by MDD appeared to be explained by co-occurring BPD symptoms, as MDD was no longer significant when both were in the model. While there are sex differences, particularly in development of sadness regulation, these sex effects do not explain the observed relationship between these trajectories and BPD.

Self-regulation and related emotion regulation commonly increase across development, are related to improved global functioning, and are inversely correlated with various forms of psychopathology (Nigg, 2017). Consistent with the idea that emotion regulation increases across development and prior work (Zimmermann & Iwanski, 2014), we saw a linear increase in coping with both anger and sadness over time, even in our high-risk sample. When assessing emotion regulation strategies, some have been identified as being maladaptive, such as inhibition or suppression, while others have been thought of as generally more adaptive, such as acceptance, reappraisal, and problem solving (for a review see Aldao et al., 2010). The Children’s Emotional Management Scale used here largely measures “effective” regulation via the coping subscale. Given the extensive literature demonstrating a relationship between use of adaptive emotion regulation and decreased rates of psychopathology transdiagnostically (see Aldao et al., 2010 for a meta-analysis), it is perhaps unsurprising that the longitudinal trajectory of coping with anger and sadness from childhood through adolescence was related to BPD, MDD, and CD symptoms. This is in keeping with prior work demonstrating emotional acceptance is associated with fewer depressive symptoms in adults (Aldao et al., 2010) and with possibly fewer depressive symptoms and aggression in adolescents (Price et al., 2022).

In contrast to the coping subscales of the CEMS, the dysregulation subscale items index dysregulated, or maladaptive, expressions of emotions. Interestingly, we found a quadratic model provided the best fit for these trajectories, where there were more dysregulated emotional expressions earlier in childhood and later in adolescence, with a decrease in between. While this may be particular to our participant group, given they were recruited for exhibiting early-onset psychopathology, we are unaware of prior studies assessing emotion dysregulation across such a wide age span with repeated assessments that allow for modeling nonlinear effects. Overall, our findings indicate that increased dysregulated expressions of negative emotions are related to increased BPD symptoms. This is consistent with a prior longitudinal study of emotion dysregulation trajectories in BPD development (Stepp, Scott, et al., 2014), which also showed that both the intercept and slope of dysregulated expressions of emotion across adolescence predicted later symptoms of BPD. Our study extends these results to a younger participant age, as our assessments of emotion dysregulation began during middle childhood. Given the emphasis on dysregulated expressions of anger in the diagnostic criteria of BPD, we hypothesized that the developmental trajectory of anger dysregulation would be specifically related to BPD. Contrary to this hypothesis, we found that while there was a relationship between both the trajectory of anger dysregulation (BPFS-C × time interaction) and a difference in initial anger dysregulation with BPD symptoms, this was not specific to BPD. Rather, MDD symptoms also corresponded to an overall difference in anger dysregulation. In contrast, only BPD symptoms explained variance in the change in sadness dysregulation across time. While this was contrary to our hypothesis, it is in keeping with prior work by Zeman et al. (2002), who showed that even when assessed separately, dysregulated expressions of both anger and sadness predicted internalizing symptoms, while dysregulated sadness was related to externalizing symptoms. Together, these findings emphasize the importance of both internalizing and externalizing components of BPD.

Prior work finds that individuals with BPD are more likely to suppress (Salsman & Linehan, 2012) or avoid negative emotions (Chapman et al., 2009; Rosenthal et al., 2005), which is in keeping with one of the hypothesized means by which invalidating parenting can impact the development of emotion regulation in BPD. As families reject the child’s emotional expressions, this rejection or suppression may then become internalized. Here, we demonstrate that expressive reluctance as assessed via the EESC is related to increased BPD symptoms over time. Additionally, while there is an increase in inhibition of both sadness and anger across childhood, there is little difference in the inhibition of anger related to BPD, MDD, or CD symptoms. Rather, elevated BPD symptoms seem related to both increased initial sadness inhibition and a steeper increase across childhood and adolescence. Again, while neither expressive reluctance nor sadness inhibition was specific to BPD, in that MDD symptoms × time also explained some of the variance in sadness inhibition and MDD symptoms explained variance in expressive reluctance, the variance accounted for by MDD symptoms appears to be primarily due to the overlap with BPD symptoms, as MDD symptoms were no longer significant when both were included in the same model.

Finally, we found poor emotional awareness to be related to BPD symptoms. While this is not specified in developmental models of BPD, there is evidence that effective emotion regulation is dependent on emotional awareness or clarity (Gratz & Roemer, 2004), which then acts as a transdiagnostic risk factor for psychopathology (Barrett et al., 2001; Kranzler et al., 2016). Several clinical theories posit that emotion dysregulation seen in BPD stems from a lack of emotional awareness (Ebner-Priemer et al., 2015; Gratz & Roemer, 2004; M. Linehan, 1993). While the quadratic trajectory of awareness over development was unexpected, given that it would appear to depend on underlying processes of internal awareness that should increase with age, it is in keeping with prior work from Kranzler et al. (2016). The authors found that in childhood and early adolescence emotional awareness was negatively associated with age, and hypothesized a possible explanation that as children move through adolescence there are increasing cognitive and social demands, perhaps taxing their emotion processing resources and decreasing emotional clarity. Such an explanation fits with the trajectory identified here and the similar nonlinear trajectory found for dysregulated expressions of emotion. The transdiagnostic nature of poor awareness is supported by our findings of a similar relationship with MDD symptoms, though again, this relationship appears at least in part due to the covariance with BPD symptoms. However, the independent relationship with BPD symptoms and prior work demonstrating a relationship between poor emotional awareness/alexithymia and self-harm and suicide ideation and behavior (Hemming et al., 2019; Iskric et al., 2020; Wolff et al., 2019), indicate further research into emotional awareness in the development of BPD specifically is important.

Indeed, a recent meta-analysis of 39 studies found a moderate, positive relationship between low emotional awareness (one component of alexithymia) and BPD (Derks et al., 2017), and other recent reviews indicate a strong relationship between alexithymia and suicide ideation and behavior and depression(Hemming et al., 2019). However, few of the studies reviewed incorporated a clinical control group, and even less included a longitudinal design. Here, we demonstrate a longitudinal, developmental relationship between low emotional awareness and BPD symptoms: poor emotional awareness across childhood and into adolescence was associated with BPD (and to a lesser extent MDD) symptoms in adolescence. Additional work indicates that alexithymia may be a crucial mediator of the relationships between childhood adversity and BPD, as well as childhood adversity and other aspects of emotion dysregulation (Edwards et al., 2021), however more longitudinal, prospective work is needed to clarify these relationships across development.

While our study has a number of particular advantages for answering the questions posed, it is not without its limitations. This prospective, 17 year longitudinal study that followed children from preschool through late adolescence has a rich database with not only repeated clinical diagnostic interviews but also multiple measures of emotional processing (Luby et al., 2014). This has allowed us to assess the unfolding trajectory of multiple components of emotion processing across an important developmental time frame and provided the opportunity to assess linear and nonlinear changes as presented here. The high rate of BPD in this group by late adolescence was unexpected, but indicates early psychopathology as a potentially important pathway in the development of BPD, as discussed in our prior work (Geselowitz et al., 2020) and by prospective studies assessing the longitudinal progression of ADHD to BPD and other personality diagnoses (Fischer et al., 2002; Miller et al., 2008; O’Grady & Hinshaw, 2023). Asking questions about the development of BPD while accounting for the presence of other forms of psychopathology is both a strength and limitation in our study. Given the specific study population, we cannot generalize these findings to development more broadly and may not be able to generalize these findings to other developmental pathways for BPD, such as impulsivity or externalizing pathways. Moreover, given this study was not designed to assess BPD, we do not have specific measures of BPD earlier in development and we are reliant on the BPFS-C, a self-report questionnaire, to identify probable BPD symptoms and diagnosis rather than using a gold standard clinical interview or collateral informants, as was done for the other psychiatric diagnoses assessed here. This also prevents us from seeing transactional relationships between the development of BPD symptoms and changes in emotion processing. While this is a relatively large study group for longitudinal studies of developmental psychopathology including deep phenotyping, it is still a modest size for detecting small effects in nonparametric analyses. While a lack of power seems less likely given the robust effects observed for BPD symptoms, it may contribute to the lack of significant effects seen for MDD and CD symptoms. Moreover, it limits our ability to explore the likely important role of sex differences in the development of emotion processing and how these interact with psychopathology.

Nonetheless, the findings presented here increase our understanding of the developmental trajectories of emotion processing and provide empirical evidence for how variations in these trajectories may impact the development of BPD, MDD, and CD. Identifying children with early-onset psychopathology who are slow to improve their emotion regulation, have ongoing high levels of both sadness and anger dysregulation, attempt to inhibit sadness, and/or have poor emotional awareness may allow us to change these trajectories and prevent or modify eventual BPD symptomatology (along with common co-occurring disorders). This also provides further support for addressing various components of emotion regulation and dysregulation with evidence-based therapies such as parent–child (Luby et al., 2018; Webster-Stratton, 2005) and dialectical behavioral therapy (DBT) based interventions (M. M. Linehan et al., 2006; McCauley et al., 2018; Perepletchikova et al., 2017).

Overall, our findings highlight the importance of the developmental trajectories of emotion regulation over time in BPD symptoms. We find a particular impact of increased dysregulation in sadness and anger, inhibition of sadness, expressive reluctance, and poor awareness. While there are transdiagnostic effects, in that many of these trajectories also impact late adolescent MDD symptoms, there are also interesting independent effects on BPD symptoms. Together, these findings support the hypothesized role for progression of emotion dysregulation as an important link in biosocial models of BPD (i.e., Crowell et al., 2009) and support addressing emotion regulation in interventions to change the trajectory of BPD symptom development and disorder onset.

## Supplementary Material

1

## Figures and Tables

**Table 1.** Participant characteristics (*n* = 187)

Demographic characteristics	%	*N*
Female gender	50.8	95
Hispanic ethnicity	3.2	6
Race		
White	54.0	101
Black	34.2	64
More than one race	11.8	22
Time 9/Time 10 assessment completion		
Time 9 only	14.4	27
Time 10 only	9.1	17
Both Time 9 and Time 10	76.5	143
**Covariates**	**Mean**	**SD**
Lifetime ACES Z-score	0.035	0.856
**Diagnostic characteristics**	**Mean**	**SD**
Mean BPFS-C total score at Time 9/10	56.0	12.9
Mean MDD core score at Time 9/10	1.47	2.07
Maximum CD sum score at Time 9/10	0.20	0.72
	**%**	***N***
BPD (as assessed by BPFS-C > 65)	35.8	67
MDD diagnosis	21.0	39
CD diagnosis	2.2	4

*Note*. ACES = adverse childhood experiences; BPD = borderline personality disorder; BPFS-C = Borderline Personality Features Scale for Children; CD = conduct disorder; MDD = major depressive disorder. BPFS-C was used to assess BPD symptoms, and putative BPD diagnosis was made via BPFS-C score above the recommended cutoff. MDD and CD symptoms were assessed via KSADS, and diagnosis was made via clinical interview.

**Table 2.** Correlations of CEMS and EESC subscales at age 10 (*N* = 137)

	1	2	3	4	5	6	7	8
1. Sadness coping	1.0000							
2. Anger coping	0.5124 *p* < 0.0001	1.0000						
3. Sadness dysregulation	− 0.1912 *p* = 0.0252	− 0.1695 *p* = 0.0477	1.0000					
4. Anger dysregulation	− 0.1957 *p* = 0.0219	− 0.4568 *p* < 0.0001	0.3214 *p* = 0.0001	1.0000				
5. Sadness inhibition	0.0090 *p* = 0.9170	− 0.1216 *p* = 0.1571	0.1314 *p* = 0.1259	0.3029 *p* = 0.0003	1.0000			
6. Anger inhibition	0.1342 *p* = 0.1180	0.3292 *p* < 0.0001	− 0.0399 *p* = 0.6438	− 0.3483 *p* < 0.0001	0.2683 *p* = 0.0015	1.0000		
7. Poor awareness	− 0.1409 *p* = 0.1018	− 0.2637 *p* = 0.0019	0.3517 *p* < 0.0001	0.4206 *p* < 0.0001	0.3948 *p* < 0.0001	0.1171 *p* = 0.1745	1.0000	
8. Expressive reluctance	− 0.1127 *p* = 0.1913	− 0.1629 *p* = 0.0581	0.2911 *p* = 0.0006	0.3349 *p* < 0.0001	0.5083 *p* < 0.0001	0.2048 *p* = 0.0168	0.6719 *p* < 0.0001	1.0000

**Table 3.** Participant ages and number of participants completing each measure at each study wave

Wave	Age		*n* Completed measure			
	Mean	SD	CEMS	EESC	BPFS-C	PAPA/CAPA/KSADS
T1	4.50	0.79				151
T2	5.51	0.79				149
T3	6.48	0.79				147
T4	9.05	0.79	137	136		137
T5	10.14	0.86	143	143		143
T6	10.92	1.06	166	166		166
T7	12.22	1.13	139	139		139
T8	13.23	1.30	80	80		80
T9	16.28	1.15	170		170	170
T10	18.55	1.20	162		160	159

*Note*. BPFS-C = Borderline Personality Features Scale for Children; CAPA = Child and Adolescent Psychiatric Assessment; CEMS = Child Emotional Management Scale; EESC = Emotion Expression Scale for Children; KSADS = Kiddie Schedule for Affective Disorders and Schizophrenia; PAPA = Preschool Age Psychiatric Assessment.

**Table 4.** Multilevel models assessing the effect of BPD symptoms on emotional processing

DV = Sadness coping	Emotion coping models					
	Estimate	SE	*t*	*p*	FDR *p*
Time	0.1477	0.0179	8.25	<0.0001	
Age at first wave	0.1946	0.0705	2.76	0.0063	
Female sex	− 0.5527	0.1623	− 3.41	0.0008	
Lifetime ACES-Z	− 0.0700	0.1051	− 0.67	0.5061	
Family affective disorder	− 0.1259	0.2067	− 0.61	0.5435	
BPD symptoms	0.0034	0.0095	0.36	0.7219	
BPD symptoms × Time	− 0.0083	0.0014	− 6.02	<0.0001	<0.0001
DV = Anger Coping	Estimate	SE	*t*	*p*	FDR *p*
Time	0.1612	0.0182	8.87	<0.0001	
Age at first wave	0.1084	0.0732	1.48	0.1402	
Female sex	0.0319	0.1715	0.19	0.8525	
Lifetime ACES-Z	− 0.0954	0.1108	− 0.86	0.3904	
Family affective disorder	0.3016	0.2183	1.38	0.1688	
BPD symptoms	− 0.0383	0.0097	− 3.95	0.0001	
BPD symptoms × Time	− 0.0047	0.0014	− 3.38	0.0009	0.0036
	**Emotion dysregulation models**				
**DV = Sadness dysregulation**	**Estimate**	**SE**	***t***	***p***	**FDR *p***
Time	− 0.2458	0.0449	− 5.47	<0.0001	
Time squared	0.0239	0.0046	5.23	<0.0001	
Age at first wave	− 0.0697	0.0513	− 1.36	0.1758	
Female sex	0.3019	0.1193	2.53	0.0123	
Lifetime ACES-Z	− 0.0301	0.0771	− 0.39	0.6966	
Family affective disorder	− 0.0939	0.1519	− 0.62	0.5373	
BPD symptoms	0.0103	0.0068	1.51	0.1337	
BPD symptoms × Time	0.0026	0.0011	2.47	0.0146	0.0234
**DV = Anger dysregulation**	**Estimate**	**SE**	***t***	***p***	**FDR *p***
Time	− 0.0910	0.0438	− 2.08	0.0382	
Time squared	0.0095	0.0045	2.13	0.0331	
Age at first wave	− 0.0133	0.0565	− 0.24	0.8136	
Female sex	− 0.1598	0.1331	− 1.20	0.2314	
Lifetime ACES-Z	0.0237	0.0859	0.28	0.7833	
Family affective disorder	− 0.2139	0.1694	− 1.26	0.2083	
BPD symptoms	0.0235	0.0073	3.21	0.0015	
BPD symptoms × Time	0.0034	0.0011	3.22	0.0015	0.0040
	**Emotion inhibition models**				
**DV = Sadness inhibition**	**Estimate**	**SE**	***t***	***p***	**FDR *p***
Time	0.1025	0.0214	4.79	<0.0001	
Age at first wave	0.0128	0.0716	0.18	0.8578	
Female sex	− 0.5389	0.1678	− 3.21	0.0016	
Lifetime ACES-Z	0.0020	0.1079	0.02	0.9853	
Family affective disorder	− 0.1390	0.2135	− 0.65	0.5157	
BPD symptoms	0.0220	0.0091	2.41	0.0168	
BPD symptoms × Time	0.0051	0.0017	3.08	0.0024	0.0048
**DV = Anger inhibition**	**Estimate**	**SE**	***t***	***p***	**FDR *p***
Time	0.0717	0.0197	3.64	0.0004	
Age at first wave	− 0.0615	0.0758	− 0.81	0.4178	
Female sex	− 0.1169	0.1798	− 0.65	0.5163	
Lifetime ACES-Z	0.0654	0.1155	0.57	0.5722	
Family affective disorder	0.0807	0.2286	0.35	0.7245	
BPD symptoms	− 0.0030	0.0075	− 0.40	0.6871	0.6871
	**Emotional expressions and understanding models**				
**DV = Poor awareness**	**Estimate**	**SE**	***t***	***p***	**FDR *p***
Time	− 3.0787	0.3831	− 8.04	<0.0001	
Time squared	0.4715	0.0893	5.28	<0.0001	
Age at first wave	− 0.7770	0.3038	− 2.56	0.0113	
Female sex	− 0.3625	0.6847	− 0.53	0.5972	
Lifetime ACES-Z	0.9412	0.4383	2.15	0.0331	
Family affective disorder	− 0.9992	0.8695	− 1.15	0.2520	
BPD symptoms	0.1214	0.0286	4.24	<0.0001	<0.0001
**DV = Expressive reluctance**	**Estimate**	**SE**	***t***	***p***	**FDR *p***
Time	− 2.2795	0.3489	− 6.53	<0.0001	
Time squared	0.4031	0.0807	4.99	<0.0001	
Age at first wave	− 0.4204	0.2625	− 1.60	0.1109	
Female sex	− 0.8825	0.5851	− 1.51	0.1333	
Lifetime ACES-Z	0.6735	0.3745	1.80	0.0738	
Family affective disorder	− 1.5077	0.7454	− 2.02	0.0446	
BPD symptoms	0.0871	0.0245	3.56	0.0005	0.0006

*Note*. ACES-Z = adverse childhood experiences Z-scored; BPD = borderline personality disorder; DV = dependent variable; FDR = false discovery rate.

**Table 5.** Effects of multi-finial psychopathology on trajectories of emotion processing

	Emotion coping models								
	IV = BPD symptoms			IV = MDD symptoms			IV = CD symptoms		
**DV = Sadness coping**	***t***	***p***	**FDR *p***	***t***	***p***	**FDR *p***	***t***	***p***	**FDR *p***
Independent variable	0.36	0.7219		1.34	0.1816		1.07	0.2865	
Independent variable × Time	− 6.02	< 0.0001	< 0.0001	− 4.13	< 0.0001	< 0.0001	− 3.18	0.0018	0.0144
**DV = Anger coping**	***t***	***p***	**FDR *p***	***t***	***p***	**FDR *p***	***t***	***p***	**FDR *p***
Independent variable	− 3.95	0.0001		− 0.81	0.4212		− 0.91	0.3617	
Independent variable × Time	− 3.38	0.0009	0.0036	− 2.36	0.0196	0.0523	− 2.95	0.0037	0.0148
**Emotion dysregulation models**									
	**IV = BPD symptoms**			**IV = MDD symptoms**			**IV = CD symptoms**		
**DV = Sadness dysregulation**	***t***	*** p***	**FDR *p***	***t***	***p***	**FDR *p***	***t***	***p***	**FDR *p***
Independent variable	1.51	0.1337		1.86	0.0647	0.0778	1.11	0.2672	0.3563
Independent variable × Time	2.47	0.0146	0.0234	–	–	–	–	–	–
**DV = Anger dysregulation**	***t***	***p***	**FDR *p***	***t***	***p***	**FDR *p***	***t***	***p***	**FDR *p***
Independent variable	3.21	0.0015		3.07	0.0024	0.0096	0.09	0.9312	
Independent variable × Time	3.22	0.0015	0.0040	–	–	–	2.55	0.0119	0.0317
**Emotion inhibition models**									
	**IV = BPD symptoms**			**IV = MDD symptoms**			**IV = CD symptoms**		
**DV = Sadness inhibition**	***t***	***p***	**FDR *p***	***t***	***p***	**FDR *p***	***t***	***p***	**FDR *p***
Independent variable	2.41	0.0168		0.88	0.3808		0.68	0.4984	0.5696
Independent variable × Time	3.08	0.0024	0.0048	3.00	0.0031	0.0124	–	–	–
**DV = Anger inhibition**	***t***	***p***	**FDR *p***	***t***	***p***	**FDR *p***	***t***	***p***	**FDR *p***
Independent variable	− 0.40	0.6871	0.6871	− 0.11	0.9154	0.9154	− 1.78	0.0768	0.2048
	**Emotional expressions and understanding models**								
	**IV = BPD symptoms**			**IV = MDD symptoms**			**IV = CD symptoms**		
**DV = Poor awareness**	***t***	***p***	**FDR *p***	***t***	***p***	**FDR *p***	***t***	***p***	**FDR *p***
Independent variable	4.24	< 0.0001	< 0.0001	2.93	0.0038	0.0101	0.40	0.6915	0.6915
**DV = Expressive reluctance**	***t***	***p***	**FDR *p***	***t***	***p***	**FDR *p***	***t***	***p***	**FDR *p***
Independent variable	3.56	0.0005	0.0006	2.27	0.0244	0.0390	1.51	0.1335	0.2136

*Note*. BPD = borderline personality disorder; CD = conduct disorder; DV = dependent variable; FDR = false discovery rate; IV = independent variable; MDD = major depressive disorder. The effect of each set of symptoms at the final two timepoints, including BPD symptoms as measured by the BPFS-C, MDD symptoms as measured using the KSADS, and CD symptoms, as measured using the KSADS on the trajectories of emotional processing including coping as measured by the CEMS-anger and -sadness coping scales, dysregulation as measured by the CEMS-anger and - sadness dysregulation scales, inhibition as measured by the CEMS-anger and -sadness inhibition scales, and poor emotional awareness and expressive reluctance as measured by the EESC. In models where the interaction between the symptom score and time was significant, these results are also included. Models covaried for age at first wave, sex, and lifetime adverse childhood experiences Z-score.
