# Supplementary material for: Developmental trajectories of anger and sadness dysregulation in childhood differentially predict later borderline symptoms
Source: Dev Psychopathol. Author manuscript; Available in PMC 2024 Dec 21. (PMC10733555; doi:10.1017/S0954579423000627)
Supplement: 1 [file NIHMS1909618-supplement-1.docx]

**Study participants were from the preschool depression study, a 17-year longitudinal student with 10 assessment waves.**

Our analyses utilized trajectories of emotion regulation ascertained from Children’s Emotional Management Scale (CEMS)^1^, which was included at assessments 4 through 10, and the Poor Awareness subscale of the Emotions Expressions Scale for Children (EESC)^2^, which was included at assessments 4 through 8. Borderline personality symptoms were assessed via the Borderline Personality Features Scale for Children (BPFS-C)^3^ at assessments 9 and 10. Symptoms of depression and conduct were assessed via standardized clinical interview. Our analyses include the 187 children who had data from the CEMS and EESC for at least one assessment and also had completed the BPFS-C at assessment 9 or 10. Differences between those children included vs excluded from analyses can be found in Table S1.

The participants who were included in our analyses did not significantly different from those who were not in emotion regulation trajectories that related to borderline symptoms. 187 children were included and 162 excluded as they either did not complete assessments 9 or 10 or did not have at least one assessment including the CEMS and EESC, which were needed to calculate the MLM intercepts and slopes. The groups did differ in anger inhibition intercept and slope, though as neither showed a significant relationship to BPFS-C score, these were not included in our analyses. There was also a statistically significant difference between groups in the sadness inhibition slope, where those included had a slightly larger increase across childhood.

|  | **Included (N=187)** | | **Excluded (N=161)** | | **Included vs. Excluded** | |
| --- | --- | --- | --- | --- | --- | --- |
|  | **%** | **N** | **%** | **N** | **χ^2^** | **p** |
| Female gender | 50.8 | 95 | 45.3 | 73 | 1.03 | 0.309 |
| Race |  |  |  |  | 0.35 | 0.838 |
| White | 54.0 | 101 | 51.5 | 83 |  |  |
| Black | 34.2 | 64 | 34.8 | 56 |  |  |
| More than one race | 11.8 | 22 | 13.7 | 22 |  |  |
|  | **Mean** | **SD** | **Mean** | **SD** | **t** | **p** |
| Baseline age | 4.50 | 0.79 | 4.40 | 0.81 | 1.04 | 0.301 |
| Overall ACES Z-score | 0.035 | 0.856 | -0.063 | 0.765 | 1.12 | 0.265 |
| Baseline MDD core score | 2.58 | 1.92 | 2.32 | 1.71 | 1.24 | 0.218 |
| Baseline CD sum score | 0.96 | 1.43 | 1.07 | 1.52 | -0.62 | 0.533 |
|  | **Included (N=187)** | | **Excluded (N=108)** | | **Included vs. Excluded** | |
|  | **Mean** | **SD** | **Mean** | **SD** | **t** | **p** |
| CEMS-Anger Inhibition intercept | 7.33 | 0.72 | 7.50 | 0.69 | -2.00 | **0.046** |
| CEMS-Anger Inhibition slope | 0.05 | 0.09 | 0.03 | 0.05 | 2.60 | **0.010** |
| CEMS-Anger Dysregulation intercept | 4.68 | 0.75 | 4.64 | 0.76 | 0.53 | 0.597 |
| CEMS-Anger Dysregulation slope | -0.01 | 0.05 | -0.01 | 0.02 | 1.18 | 0.240 |
| CEMS-Anger Coping intercept | 9.00 | 0.97 | 9.08 | 0.89 | -0.74 | 0.459 |
| CEMS-Anger Coping slope | 0.16 | 0.07 | 0.15 | 0.03 | 0.54 | 0.590 |
| CEMS-Sadness Inhibition intercept | 7.77 | 0.71 | 7.84 | 0.60 | -0.84 | 0.402 |
| CEMS-Sadness Inhibition slope | 0.08 | 0.12 | 0.06 | 0.05 | 2.39 | **0.018** |
| CEMS-Sadness Dysregulation intercept | 4.92 | 0.51 | 4.92 | 0.47 | -0.04 | 0.969 |
| CEMS-Sadness Dysregulation slope | -0.03 | 0.06 | -0.04 | 0.03 | 1.48 | 0.141 |
| CEMS-Sadness Coping intercept | 11.22 | 0.73 | 11.21 | 0.61 | 0.09 | 0.927 |
| CEMS-Sadness Coping slope | 0.14 | 0.06 | 0.14 | 0.02 | 0.27 | 0.785 |
| EESC Poor Awareness intercept | 19.50 | 3.01 | 20.06 | 2.90 | -1.57 | 0.118 |
| EESC Poor Awareness slope | -1.28 | 0.71 | -1.30 | 0.64 | 0.27 | 0.786 |

*Table S1. Characteristic of participants included vs excluded from analyses*

**There are similar relationships between the trajectories of emotion regulation and development of categorical BPD diagnosis as with development of BPD symptoms.**

***The development of emotion regulation of sadness and anger across childhood along with overall emotional awareness predicts later BPD symptoms.***

When considered independently, both the intercept and slopes of CEMS-Anger dysregulation and coping as well as CEMS-Sadness inhibition and coping predicted later BPD categorical diagnosis (BPFS-C > 65), covarying for ACES-Z and ever having a diagnosis of MDD (Table S2).

|  | **Estimate** | **SE** | **r^2^** | **p** | **FDR p** |
| --- | --- | --- | --- | --- | --- |
| CEMS-Anger Dysregulation intercept | 0.69 | 0.24 | 8.63 | 0.0033 | 0.0061 |
| CEMS-Anger Dysregulation slope | 12.93 | 3.83 | 11.37 | 0.0007 | 0.0019 |
| CEMS-Anger Coping intercept | -0.81 | 0.20 | 16.53 | <0.0001 | <0.0001 |
| CEMS-Anger Coping slope | -10.73 | 2.68 | 16.07 | <0.0001 | <0.0001 |
| CEMS-Sadness Inhibition intercept | 0.52 | 0.24 | 4.61 | 0.0318 | 0.0350 |
| CEMS-Sadness Inhibition slope | 3.93 | 1.49 | 7.00 | 0.0082 | 0.0129 |
| CEMS-Sadness Dysregulation intercept | 0.57 | 0.33 | 3.01 | 0.0827 | 0.0827 |
| CEMS-Sadness Dysregulation slope | 7.91 | 3.06 | 6.70 | 0.0096 | 0.0132 |
| CEMS-Sadness Coping intercept | -0.75 | 0.25 | 9.01 | 0.0027 | 0.0059 |
| CEMS-Sadness Coping slope | -18.99 | 3.98 | 22.80 | <0.0001 | <0.0001 |
| EESC Poor Awareness intercept | 0.14 | 0.06 | 5.96 | 0.0147 | 0.0180 |

*Table S2. Logistical Regression of BPFS-C Score > 65 at Assessments 9 or 10 by Intercepts and Slopes from MLM’s of CEMS and EESC Subscales.*

*All analyses covary for overall ACES Z-Score and ever having a diagnosis of MDD at Assessments 1-10.*

***There are different relations of coping, dysregulation, and inhibition of anger and sadness to later BPD diagnosis.***

We next examined independent relationships of each type of anger regulation to BPD by entering anger dysregulation and coping in the same model, controlling for MDD diagnosis and ACES. Anger coping but not dysregulation intercept independently predicts later BPD diagnosis, while both anger coping and dysregulation slope have independent contributions to BPD diagnosis. There was no significant relationship between sadness dysregulation intercept and BPD diagnosis, while sadness coping intercept predicted BPD diagnosis, however, both sadness coping and dysregulation slopes were independently related to later diagnosis of BPD (Table S3).

| **Models assessing independent effects of dysregulation, coping, and inhibition** | | | **est** | | | | **t** | **FDR p** | | | |
| --- | --- | --- | --- | --- | --- | --- | --- | --- | --- | --- | --- |
| Anger | Dysregulation | intercept | 0.13 | | | 0.19 | | | 0.660 | | |
| **Anger** | **Coping** | **intercept** | -0.75 | | | 9.39 | | | **0.004** | | |
| **Anger** | **Dysregulation** | **slope** | 9.15 | | | 5.17 | | | **0.023** | | |
| **Anger** | **Coping** | **slope** | -9.18 | | | 10.56 | | | **0.002** | | |
| **Sadness** | **Dysregulation** | **slope** | 8.89 | | | 6.78 | | | **0.009** | | |
| **Sadness** | **Coping** | **slope** | -19.98 | | | 22.75 | | | **<0.001** | | |
|  | | | **est** | | | | **t** | | | **FDR p** | |
| **Anger** | **Dysregulation** | **intercept** | 0.71 | | 8.36 | | | | | **0.004** | |
| **Anger** | **Dysregulation** | **slope** | 12.65 | | 11.09 | | | | | **0.002** | |
| **Anger** | **Coping** | **intercept** | -0.82 | | 16.16 | | | | | **<0.001** | |
| **Anger** | **Coping** | **slope** | -10.79 | | 15.58 | | | | | **<0.001** | |
| **Sadness** | **Coping** | **intercept** | -0.72 | | 7.24 | | | | | **0.007** | |
| **Sadness** | **Coping** | **slope** | -18.50 | | 21.70 | | | | | **<0.001** | |
| Sadness | Inhibition | intercept | 0.44 | | 3.17 | | | | | 0.075 | |
| **Sadness** | **Inhibition** | **slope** | 3.58 | | 5.65 | | | | | **0.035** | |
|  | | | **est** | **t** | | | | | | | **FDR p** |
| **Anger** | **Coping** | **intercept** | -0.71 | | 9.05 | | | | | **0.005** | |
| Sadness | Coping | intercept | -0.22 | | 0.51 | | | | | 0.474 | |
| **Anger** | **Coping** | **slope** | -6.10 | | 4.19 | | | | | **0.041** | |
| **Sadness** | **Coping** | **slope** | -15.61 | | 13.46 | | | | | **<0.001** | |
| **Anger** | **Dysregulation** | **slope** | 11.26 | | 8.12 | | | | | **0.009** | |
| Sadness | Dysregulation | slope | 5.75 | | 3.15 | | | | | 0.076 | |

*Table S3. Logistic Regressions of BPFS-C Total >65 at Assessments 9 or 10 by intercepts and slopes from the CEMS emotion regulation scales when assessed together. Each box denotes an individual model.*

*All analyses covary for overall ACES Z-Score and diagnosis of MDD at Assessments 1-10.*

*Results that remain significant after correcting for multiple comparisons are in bold.*

***The independent relationships of intercept and slope to BPD diagnosis differed both by type of emotion and measure of regulation.***

We find similar results when predicting categorical BPD (BPFS-C >65) as with BPFS-C score, including when controlling for ACES-Z and diagnosis of MDD. Anger coping intercept and slope and sadness coping intercept and slope all independently predict later BPD diagnosis when slope and intercept were included in the same model. However, when sadness and anger were considered together, only the anger coping intercept contributed to BPD diagnosis. Both anger and sadness coping slopes independently predicted later BPD diagnosis when assessed together. Only anger dysregulation slope independently predicted BPD diagnosis, while sadness dysregulation slope was not an independent predictor when assessed together. Sadness inhibition intercept and slope both independently predicted categorical BPD diagnosis. See Table S3.

***While the development of some aspects of emotion regulation also predicted MDD and CD psychopathology, the broadest relationships are with Borderline diagnosis.***

When considering categorical diagnosis of BPD, MDD, and CD, similar patterns emerge. As above, all scales except sadness dysregulation intercept predict categorical BPD. Only anger dysregulation, sadness inhibition, and sadness coping slopes, as well as levels of poor awareness at age 10 are predictive of categorical MDD. There is no statistical difference in the ability to predict BPD vs MDD diagnosis as measured by Steiger’s Z for these scales. Only anger dysregulation and coping slopes predicted categorical CD. For both, there is no statistical difference in the ability to predict BPD vs CD.

**Zero-inflated Poisson regression models were used to assess the relationship between emotion regulation and conduct symptoms.**

Given the low rates of CD symptoms (168/187 participants with 0 symptoms), zero-inflated Poisson regression models were also used to assess the relationship between emotion regulation and CD symptoms. Greater anger dysregulation slope was found to be predictive of greater number of CD symptoms in those with non-zero CD symptoms, while a smaller anger coping slope was found to be predictive of having a non-zero CD symptoms score, but not predictive of the number of symptoms when present. Using these Poisson models, greater anger dysregulation intercept, smaller anger coping intercept, and greater poor emotional awareness intercepts also predicted having a non-zero number of CD symptoms, greater sadness dysregulation slope predicted a greater number of CD symptoms when non-zero, and a smaller sadness coping slope predicted both having non-zero CD symptoms and greater number of symptoms when non-zero. However, none of these survived FDR correction for multiple comparisons (table S4).

|  | **Estimate** | **SE** | **t** | **p** | **FDRp** |
| --- | --- | --- | --- | --- | --- |
| *CEMS-Anger Dysregulation intercept* |  |  |  |  |  |
| Zero-inflated: no CD symptoms | -0.88 | 0.40 | -2.18 | **0.030** | 0.101 |
| Continuous: number of CD symptoms | -0.08 | 0.32 | -0.26 | 0.798 | 0.871 |
| *CEMS-Anger Dysregulation slope* |  |  |  |  |  |
| Zero-inflated: no CD symptoms | -4.80 | 5.29 | -0.91 | 0.364 | 0.364 |
| Continuous: number of CD symptoms | 7.24 | 2.94 | 2.46 | **0.014** | 0.132 |
| *CEMS-Anger Coping intercept* |  |  |  |  |  |
| Zero-inflated: no CD symptoms | 0.71 | 0.35 | 2.04 | **0.042** | 0.101 |
| Continuous: number of CD symptoms | -0.45 | 0.28 | -1.59 | 0.111 | 0.333 |
| *CEMS-Anger Coping slope* |  |  |  |  |  |
| Zero-inflated: no CD symptoms | 9.73 | 4.05 | 2.40 | **0.016** | 0.101 |
| Continuous: number of CD symptoms | -4.12 | 2.81 | -1.46 | 0.143 | 0.343 |
| *CEMS-Sadness Inhibition intercept* |  |  |  |  |  |
| Zero-inflated: no CD symptoms | -0.77 | 0.42 | -1.85 | 0.064 | 0.128 |
| Continuous: number of CD symptoms | -0.10 | 0.33 | -0.29 | 0.772 | 0.871 |
| *CEMS-Sadness Inhibition slope* |  |  |  |  |  |
| Zero-inflated: no CD symptoms | -2.72 | 2.51 | -1.09 | 0.278 | 0.303 |
| Continuous: number of CD symptoms | -1.03 | 2.33 | -0.44 | 0.659 | 0.871 |
| *CEMS-Sadness Dysregulation intercept* |  |  |  |  |  |
| Zero-inflated: no CD symptoms | -0.91 | 0.58 | -1.55 | 0.121 | 0.182 |
| Continuous: number of CD symptoms | -0.16 | 0.53 | -0.29 | 0.762 | 0.871 |
| *CEMS-Sadness Dysregulation slope* |  |  |  |  |  |
| Zero-inflated: no CD symptoms | 7.18 | 5.86 | 1.23 | 0.221 | 0.275 |
| Continuous: number of CD symptoms | 9.70 | 4.41 | 2.20 | **0.028** | 0.132 |
| *CEMS-Sadness Coping intercept* |  |  |  |  |  |
| Zero-inflated: no CD symptoms | 0.50 | 0.37 | 1.35 | 0.178 | 0.237 |
| Continuous: number of CD symptoms | -0.10 | 0.27 | -0.36 | 0.717 | 0.871 |
| *CEMS-Sadness Coping slope* |  |  |  |  |  |
| Zero-inflated: no CD symptoms | 9.68 | 4.75 | 2.04 | **0.041** | 0.101 |
| Continuous: number of CD symptoms | -5.86 | 2.74 | -2.14 | **0.033** | 0.132 |
| *EESC Poor Awareness intercept* |  |  |  |  |  |
| Zero-inflated: no CD symptoms | -0.23 | 0.10 | -2.34 | **0.019** | 0.101 |
| Continuous: number of CD symptoms | -0.09 | 0.06 | -1.33 | 0.183 | 0.366 |
| *EESC Poor Awareness slope* |  |  |  |  |  |
| Zero-inflated: no CD symptoms | -0.56 | 0.34 | -1.65 | 0.100 | 0.171 |
| Continuous: number of CD symptoms | -0.01 | 0.20 | -0.06 | 0.953 | 0.953 |

***Supplementary Table 4.*** **Zero-Inflated Poisson Regression Models of Maximum CD Sum Score at Assessments 9/10 by CEMS and EESC Intercepts and Slopes.** FDR correction for multiple comparisons performed for zero-inflated and continuous models separately. *CD = conduct disorder.*
